# Investing in Threatened Species Conservation: Does Corruption Outweigh Purchasing Power?

**DOI:** 10.1371/journal.pone.0022749

**Published:** 2011-07-27

**Authors:** Stephen T. Garnett, Liana N. Joseph, James E. M. Watson, Kerstin K. Zander

**Affiliations:** 1 Research Institute for Environment and Livelihoods, Charles Darwin University, Darwin, Northern Territory, Australia; 2 Global Conservation, Wildlife Conservation Society, The Bronx, New York, United States of America; 3 The Ecology Centre, University of Queensland, St Lucia, Queensland, Australia; University of Western Ontario, Canada

## Abstract

In many sectors, freedom in capital flow has allowed optimization of investment returns through choosing sites that provide the best value for money. These returns, however, can be compromised in countries where corruption is prevalent. We assessed where the best value for money might be obtained for investment in threatened species that occur at a single site, when taking into account corruption. We found that the influence of corruption on potential investment decisions was outweighed by the likely value for money in terms of pricing parity. Nevertheless global conservation is likely to get best returns in terms of threatened species security by investing in “honest” countries than in corrupt ones, particularly those with a high cost of living.

## Introduction

In 2008 the operating expenditure of the four largest international conservation organizations topped US$1 billion: The Nature Conservancy $616 million [1], Wildlife Conservation Society $205 million [2], WWF International $161 million [3], Conservation International $144 million [4]. While this is only a fraction of what is needed [5], [6], the total is substantial when considered together with the many other government, non-government and business investments in conservation. This can have a substantial benefit for local economies, particularly in rural and remote areas where many of the world's poor coexist with conservation assets. In many ways, therefore, foreign investment (FDI) for conservation investment might be expected to operate along lines similar to other FDI.

Multi-country studies of FDI suggest that investment flows are influenced first by the presence of assets, such as natural resources or human capacity. Given the presence of such assets, decisions are then based around a range of financial and governance considerations such as cost of labour, tax concessions, government stability, internal and external conflict, corruption and ethnic tensions, law and order, democratic accountability of government, and quality of bureaucracy [7]. Such motivations resemble the criteria for prioritising investment in conservation assets [8], [9]: providing greatest support to the most threatened conservation values and supporting conservation in countries where the likelihood of success is highest, as evidenced by factors such as strong political support for conservation [10], supportive legislation and enforcement [11], low corruption and matching funding at appropriate levels [12].

While there has been a significant push to start incorporating cost into conservation plans [13]–[16], no study to our knowledge has simultaneously considered the cost-effectiveness of conservation decisions and the consequences of corruption costs. Corruption manifests itself in various ways including embezzlement of funds, demanding of bribes for compliance, patronage or political influence and acceptance of bribes to overlook illegal activities [17]. This can reduce the effectiveness of conservation programs by reducing the financial resources, law enforcement and political support available for conservation [18] as well as acting as an incentive for the overexploitation of resources [19] and delaying environmental recovery [20]. Effectively corruption can stifle effective investment in conservation just as it does investment in economic growth [21]. It is also seen as one of the major impediments to conservation in tropical countries [22], [23]. However, poor countries can offer a better return on investment than those with a high cost of living [24]. And although there is a strong correlation [25], poor countries are not necessarily corrupt nor are rich countries honest. Just as the freedom of movement of global capital has encouraged investment in countries with low labor and other costs [26], [27], so global conservation capital can potentially receive greater dividends in terms of effective management through investment in poorer countries.

More Endemic Bird Areas, biodiversity hotspots and other high priority terrestrial eco-regions occur in countries containing lower governance scores than in countries without such conservation assets [28]. Single site threatened species (SSTS), the 20% of the 4,239 threatened mammals, birds, tortoises and turtles, and amphibians listed by the IUCN that are dependent for their survival on conservation at single sites in the short- to medium term [29], are more evenly spread around the globe. This gives a wider choice for potential investments making it possible to maximize efficiency of conservation investment, although such investment choices could require trade-offs that may include extinction [30] if funds are insufficient. All SSTS live in places that are irreplaceable. Thus minimizing costs by optimizing choice of sites [31] is not possible.

In this paper we explore the trade-offs between corruption and financial return on conservation investment for single site threatened species. We also explore the influence of potential conservation cost on efficient investment decisions, recognising that some species are more expensive to conserve than others but considering all to be equally worthy of conservation.

## Results

**Figure 1:**
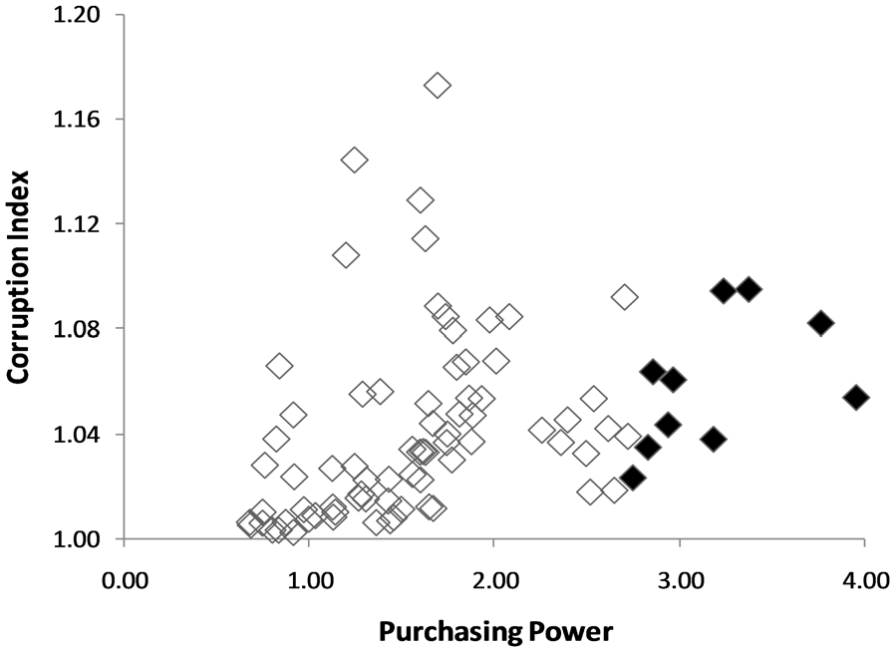
Corruption Index and Purchasing Power Parity for countries with Single Site Threatened Species (filled markers indicate the ten countries giving greatest returns on investment).

The choice of country in which to invest funds for conserving single site threatened species (SSTS) varied substantially depending on the relative influence of number of SSTS, purchasing power parity (PPP) or the potential for corruption on investment decisions. Predictably the cheapest and most corrupt are largely very poor while the more expensive but more honest are relatively wealthy, or are possessions of wealthy nations. However the ten countries that rank highest when both corruption and purchasing power are considered are all such poor nations that the value of the dollar renders corruption affordable (Table 1). These ten lie along the right side of the corruption/purchasing power data cloud (Figure 1). The level of corruption affects the investment priorities only when the number of SSTS present in the country is considered. Thus, among the 23 countries with a single SSTS, Ghana ranks higher than many poorer countries because of its relative honesty. Similarly, among the ten countries ranked highest for number of SSTS, New Zealand, with the lowest level of corruption, ranks highest because, though relatively expensive, it has the best corruption index score of any country.

**Table 1:** Highest and lowest ranking countries for investment in Single Site Threatened Species prioritized against different criteria.

Most species	Cheapest	Most honest	Best value	Best value including species no.
Mexico	Ethiopia	New Zealand	Ethiopia	Mexico
Colombia	Pakistan	Canada	Pakistan	Colombia
Peru	Guinea	Australia	Guinea	Brazil
Indonesia	Kyrgyzstan	Gough, Inaccessible, Henderson Is. (UK)	Sao Tome and Principe	Australia
Brazil	Sao Tome and Principe	Amsterdam I. (France)	Kyrgyzstan	United States of America
Cuba	Iran	Chile	Vietnam	Peru
China	Vietnam	Bermuda	Iran	Indonesia
Ecuador	Uganda	Japan	India	Japan
United States of America	India	St Lucia	Ghana	China
Madagascar	Ghana	United States of America	Uganda	New Zealand

Fewest species (1 each)	Most expensive	Most corrupt	Worst value	Worst value including species no.
Amsterdam I. (France), Angola, Armenia, British Virgin Is., Cape Verde, Cook Is., Djibouti, Dominica, El Salvador, Equatorial Guinea, Ethiopia, Fiji, Ghana, Grenada, Israel, Italy, Kyrgyzstan, Le Reunion, Oman, Pakistan, Samoa, Sri Lanka, Turkey	Japan	Somalia	Japan	Ethiopia
	Gough, Inaccessible, Henderson Is. (UK)	Equatorial Guinea	Gough, Inaccessible, Henderson Is. (UK)	Pakistan
	Le Reunion	Zimbabwe	Northern Marianas	Kyrgyzstan
	Amsterdam I. (France)	Papua New Guinea	Le Reunion	Ghana
	Northern Marianas	Angola	Amsterdam I. (France)	Sri Lanka
	Australia	Guinea	New Caledonia	Armenia
	French Polynesia	Kyrgyzstan	French Polynesia	El Salvador
	Canada	Venezuela	Australia	Djibouti
	New Caledonia	Cote d'Ivoire	Canada	Dominica
	Bermuda	Haiti	Bermuda	Turkey

Ranks are best to worst in top section, worst to best in lower section. Best value including species number sorts the countries with the most and least SSTS taking account of PPP and CI.

**Figure 2:**
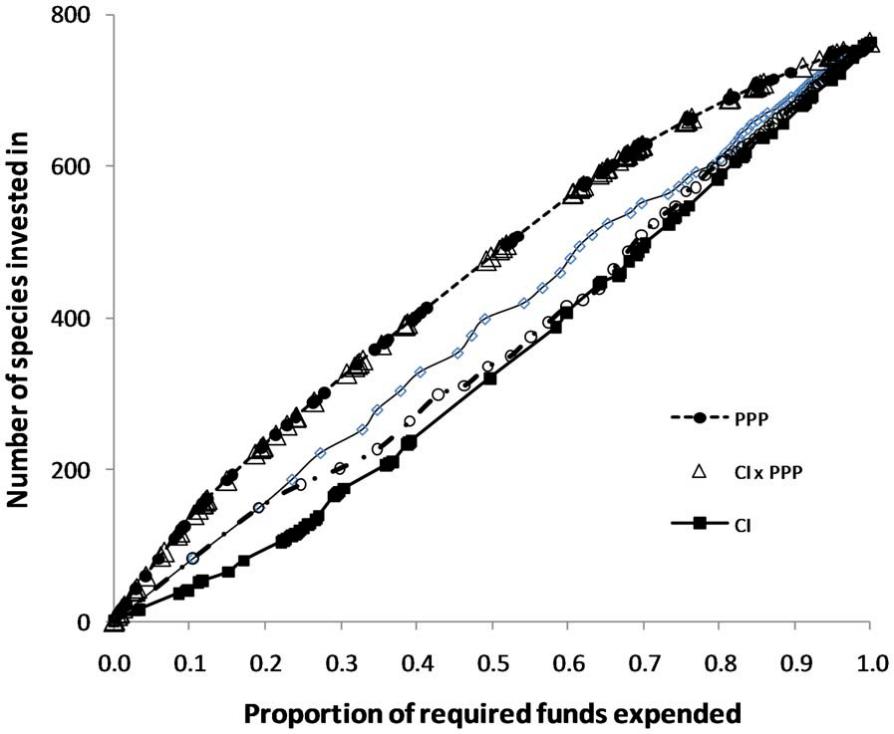
Cumulative number of Single Site Threatened Species (SSTS) prioritised on the basis of number of species (n); purchasing power parity (PPP); corruption index (CI), CI*PPP, CI*PPP*n against the proportion of the total funds required to maintain all SSTS ((n*CI*PPP)/Σ(n*CI*PPP)). PPP and CI*PPP are virtually overlapping so only symbols are presented for CI*PPP.

**Figure 3:**
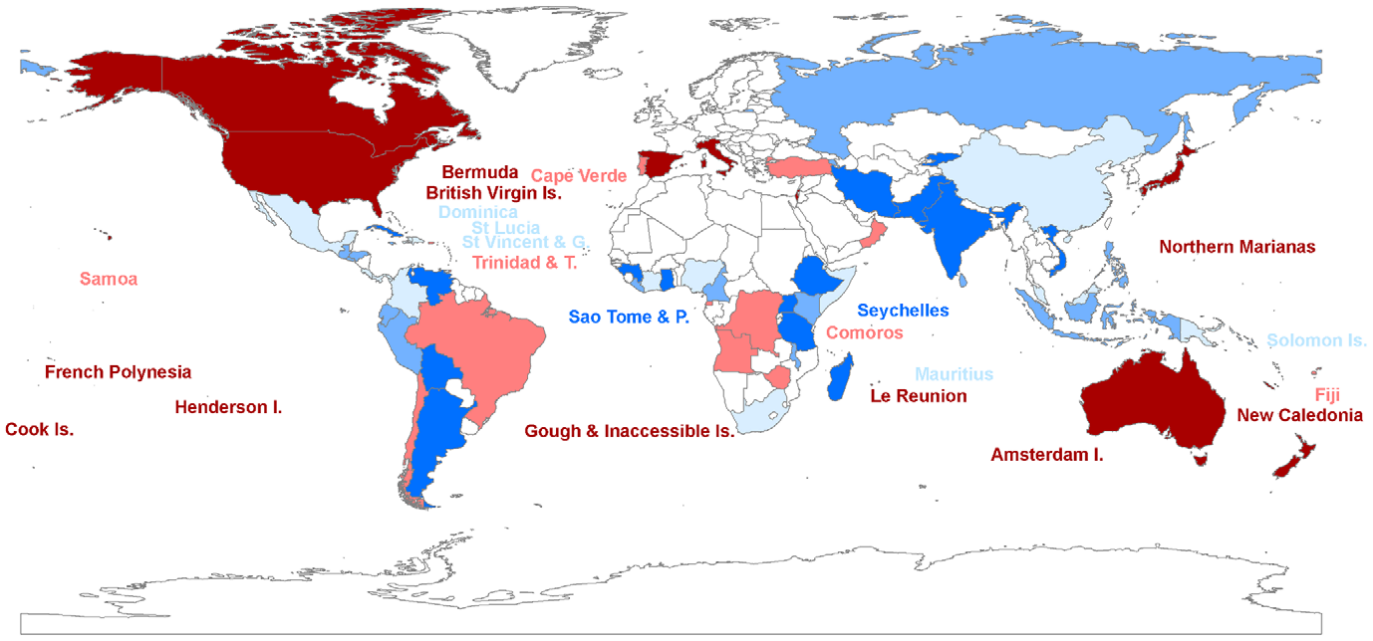
Map of priorities for funding of Single Site Threatened Species (SSTS) based on the balance between the purchasing power parity and the corruption index. Quintile colours run from dark blue (high returns on investment) through light blue, pink and red to crimson (low returns on investment). Countries in white lacked analysed SSTS.

We found that a strategy that prioritizes investment solely on the basis of the purchasing power of the dollar accumulates conservation investment rapidly whereas one that minimizes losses to corruption has a lower accumulation rate that is closely associated with the number of SSTS in a country (Figure 2). If only half the required funds are available, 349 species across 36 countries will have been managed (i.e. threats ameliorated to enhance probability of persistence) if corruption minimization is used as the main priority for funding whereas 498 species in 43 countries will have been managed if value for money is the sole criteria (Table 2). When the number of SSTS in a country is the only driver of investment, the returns on investment rise steadily on a trajectory between the other two because the number of SSTS is spread among countries with a variety of corruption index and purchasing power parity (PPP) scores. Because value of the dollar is so much more influential than corruption on potential investment strategies, the efficient strategy that balances corruption index and PPP is virtually indistinguishable from PPP alone. Thus the countries that would ostensibly give the greatest returns on investment in SSTS based on the value of the dollar after corruption are also considered among the poorest in the world (Figure 3).

**Table 2:** The proportion of countries receiving Single Site Threatened Species (SSTS) investments based on five investment strategies, assuming the same average cost of species management.

Disbursement criteria	Most species	Cheapest	Most honest	Best value	Best value including species no.
% SSTS in top ten countries	377	44	105	44	350
% SSTS in top 50% countries	90	52	42	51	89
% countries receiving investment if 50% of required funds available	13	44	48	52	11

## Discussion

Conservation FDI by non-government organizations and others needs to be based on sound business principles if donors' funds are to be effective and their influence sustained. The two factors explored here, corruption and purchasing power parity, are examples of considerations that have to be made before investment occurs. Many other sustainability, equity or cost efficiency measures could be used in a similar manner to prioritize reserve acquisition, carbon retention or other conservation investments. Regardless of the conservation objective, the message is that risk associated with conservation FDI needs to be managed in the same way as that of other direct investment, even if the criteria for success of a venture might differ. For the example used here, single-site threatened species, our analyses suggest that prioritizing primarily on the basis of the potential for corruption is much less efficient than doing so on the basis of value for money. This result is supported by earlier analyses [32] with respect to protected area conservation, and is also consistent with business literature where country-specific direct costs of production are but one of a constellation of factors affecting investment [32], [33]. This finding does not mean that all available SSTS funds should be spent in low income countries but that corruption levels should be less influential than PPP in any broader risk assessment.

The results suggest that investment in the highest risk countries is warranted despite prevailing levels of corruption. The map in Figure 3 looks very different to prioritization maps based around hot spots or other combinations of threat and biodiversity [34]. While it could be argued that losses to bribery of local officials is just one aspect of corruption, with delays, opportunity costs, transaction costs associated with operating in the underground economy and uncertainty of returns on investments all adding to investment disincentives, the actual funds lost to corruption, even if bribes are paid, are relatively low compared to differences in wealth between nations.

This too reflects business decisions where investment in high value resources, such as oil, coltan or diamonds, occurs despite corruption. In fact some studies have shown that a certain level of corruption increases FDI because of increased efficiency within bureaucracies [35], though other studies of the same countries over a longer period showed that corruption inhibited both growth and investment [36]. It is thus incumbent on investors to adopt corruption management strategies rather than try to avoid corruption altogether.

How this is done is potentially a rich avenue of research that can also draw on the economic and development literature [37], [38]. In particular the more Machiavellian strategies of companies extracting finite resources over a short time period, such as bribes and mercenaries, need to be contrasted with those of companies wishing to develop a market that can provide sustained profits over extended periods. Strategies which deter corruption, such as payment of fair wages, more stringent accounting procedures and management partnerships, need to be deployed in countries with low governance scores [25], [28]. However the existence of species that will no longer be available for investment without immediate intervention may make corruption tempting, even if their long-term maintenance, the ultimate objective of conservation, will eventually demand an entirely different approach. Perhaps conservation investment should be more closely coupled to the free trade arguments which, despite widespread criticism, have reduced poverty [39], and improved social function and governance [40] – linking conservation FDI to a raft of reforms that reduce investment inefficiencies, taking into account country-specific negative and positive externalities that will affect conservation decisions. Certainly the wrong message will be transmitted if conservation investors reward corrupt countries simply because they are more effective at threatening their biodiversity.

In this respect corruption, as well as value for money in terms of pricing parity, could still usefully be added to some of the analyses of cost-effectiveness for global prioritization. For example, Madagascar, Papua New Guinea, Cuba, Indonesia and Brazil are listed as the five countries most poorly funded for the conservation of mammals in proportion to the cost of conservation [31]. Cuba, however, is less than half as corrupt as Papua New Guinea and therefore may be a much better country in which to undertake conservation. The principal message, however, is that discrepancies in wealth, of which corruption can be a symptom as well as a cause, have the greatest potential influence on efficiency in SSTS investment. Arguably investment in the least wealthy countries with SSTS could also maximize the social benefit of threatened species investment.

## Materials and Methods

Countries supporting SSTS were identified from the database of the Alliance for Zero Extinction [41]. Species listed as other than Critically Endangered (CR), Endangered (EN) or Vulnerable (VU) were excluded, leaving 764 species. For each of the 85 countries included in the analysis, the PPP was determined from the ratio of the PPP conversion factor (the number of units of a country's currency required to buy the same amount of goods and services in the domestic market as a $US would buy in the United States [42]) and the real exchange rate between each country's currency and the $US (as at 14 April 2010), providing the cost of the bundle of goods that make up gross domestic product (GDP) across countries (i.e. dollars needed to buy a dollar's worth of goods in the country as compared to the United States). At the time of data collection the purchasing power parity (PPP), which was standardized to the value of the US$, varied from $3.98 (Ethiopia) to $0.67 (Japan). It was thus assumed that each dollar spent on conservation action in Ethiopia, the least expensive country, could purchase just $0.17 worth of conservation action in Japan. Estimates of the money lost to corruption were based on World Bank estimates of the percentage of revenues firms pay in unofficial payments per annum to public officials [43]. This data is categorical (% firms paying <1%, 1–2%, 2–10%, 10–12%, 13–25%, >25%) which was converted to a single figure by summing the product of the maximum for each category and the percentage of firms paying in that category (the category >25% was taken as 50%, but made no difference if taken as 100% as an average of only 1% of firms paid bribes of this size). As the relevant information was only available for 58 countries, the percentage of revenue scores were correlated with governance measures for the same countries using the Control of Corruption Index of the World Bank [44]. The best fit was y = 0.2203e^−5016x^ (R^2^ = 0.5428). We did also test the bribery estimates against a range of global datasets on governance and human development [44], [45] using multiple OLS regression models, GLM and mixed-effects models. We tested for interactions between the explanatory variables and applied the stepwise function in the program R to obtain the best model using the AIC. However, while we were able to develop a linear regression model with an adjusted R^2^ of 0.592 in which the significant variables were the World Bank indicators Rule of Law, Voice and Accountability, Regulatory Quality, and Control of Corruption, and the UNDP indicators GDP per capita, PPP, Human Development Index, Life expectancy at birth and Mean years of schooling of adults, several of these variables were significant in unexpected directions and we felt that the simpler exponential relationship between the average amounts paid in bribes and the Control of Corruption Index was probably as likely to give accurate estimates of the missing values as the complex model. We were also aware that, regardless of the regression analysis used, the variation in buying power was over 30 times greater than the variation in the effects of CI so that minor variations in the missing values were unlikely to influence the final result.

This equation was then used to translate the Corruption Perception Index scores of the countries with single site threatened species that lacked World Bank estimates of revenue loss (41 countries supporting 38% of SSTS considered) into an approximation of the proportion of each dollar spent that reached its conservation target after bribes had been paid. Resulting estimates varied from 0.1% average loss for New Zealand to 7.6% for Somalia. For French Polynesia (France), Amsterdam (France), Gough (UK), Inaccessible (UK) and Henderson Islands (UK) (collectively supporting 2% of SSTS) information on PPP and corruption was derived from the relevant colonial nation with a nominal 20% surcharge on PPP to account for the higher costs of investment arising from isolation. For the British Virgin Islands (supporting 1 SSTS), which lacked any estimate of corruption, data from the American Virgin Islands were used).

The product of PPP and the corruption index was used to estimate the interaction between the two: a dollar spent on conservation in a country in which US$1 buys two units of conservation but with a corruption index of 0.5 would have the same impact on the ground as a dollar spent in the US assuming it had no corruption (CI = 1.0).

Following the argument of Balmford et al. [5], one SSTS was deemed to cost, on average, the equivalent to maintain in local currency regardless of country. On this basis countries were ranked using four different metrics to guide alternative investment strategies within different financial risk environments:

1. Number of SSTS/country (n): the top priority for investment is the one with the most SSTS.
2. Purchasing Power Parity (PPP): the investment strategy aims to gain greatest value for money, regardless of number of species or the level of corruption
3. Corruption Index (CI): the investment strategy aims to avoid rent seeking behavior, regardless of other considerations
4. Efficient (PPP×CI): the investment strategy aims to optimize investment, balancing the value of the dollar against levels of corruption.
  In addition the ten countries with the highest SSTS and the 23 with only one SSTS were ranked based on the efficient investment estimates.
5. Maximized: the investment strategy aims to maximize the number of species after value of the dollar and corruption risk has been taken into account.

Using a sequential investment strategy (i.e. all SSTS in one country will be invested in before any in the next), the cumulative total of species and the cumulative total conservation units expended (n×PPP×CI for each country, standardized to total 1.00) were calculated for each ranking strategy.

## References

[1] The Nature Conservancy (2009). The Nature Conservancy Consolidated Financial Statements as of June 30 2009 and 2008.

[2] Wildlife Conservation Society (2008). Annual Report.

[3] World Wildlife Fund International (2008). Annual Report 2008 - Funding and Financial Overview.

[4] Conservation International (2008). Conservation International Foundation and Affiliates. Consolidated Financial Report June 30, 2009.

[5] Balmford A, Gaston KJ, Blyth S, James A, Kapos V (2003). Global variation in terrestrial conservation costs, conservation benefits, and unmet conservation needs.. Proc Natl Acad Sci U S A.

[6] James AN, Gaston KJ, Balmford A (1999). Balancing the Earth's accounts.. Nature.

[7] Busse M, Hefeker C (2007). Political risk, institutions and foreign direct investment.. Eur J Polit Econ.

[8] Wilson KA, McBride MF, Bode M, Possingham HP (2006). Prioritizing global conservation efforts.. Nature.

[9] Wilson KA, Underwood EC, Morrison SA, Klausmeyer KR, Murdoch WW (2007). Conserving Biodiversity Efficiently: What to Do, Where, and When.. PLoS Biol.

[10] Myers N (1988). Lifting the veil on perverse subsidies.. Nature.

[11] Leader-Williams N, Harrison J, Green MJB (1990). Designing protected areas to conserve natural resources.. Sci Prog.

[12] Bruner AG, Gullison RE, Balmford A (2004). Financial Costs and Shortfalls of Managing and Expanding Protected-Area Systems in Developing Countries.. Bio Science.

[13] Possingham HP, Andelman SJ, Noon BR, Trombulak S, Pulliam HR, Orians G, Soulé M (2001). Making Smart Conservation Decisions.. Research Priorities for Conservation Biology.

[14] Bode M, Watson J, Iwamura T, Possingham HP (2008). The cost of conservation.. Science.

[15] McCarthy MA, Thompson CJ, Garnett ST (2008). Optimal investment in conservation of species.. J Appl Ecol.

[16] Joseph LN, Maloney RF, Possingham HP (2009). Optimal Allocation of Resources among Threatened Species: a Project Prioritization Protocol.. Conserv Biol.

[17] Davis J (2004). Corruption in public service delivery: experience from South Asia's water and sanitation sector.. World Devel.

[18] Damania R, Fredriksson PG, List JA (2003). Trade liberalization, corruption, and environmental policy formation: theory and evidence.. J Environ Econ Manag.

[19] Smith RJ, Walpole MJ (2005). Should conservationists pay more attention to corruption?. Oryx.

[20] López R, Mitra S (2000). Corruption, Pollution, and the Kuznets Environment Curve.. J Environ Econ Manag.

[21] Wei S-J (2000). Local Corruption and Global Capital Flows.. Brookings Pap Eco Ac.

[22] Ceballos G, Vale MM, Bonacic C, Calvo-Alvarado J, List R (2008). Conservation Challenges for the Austral and Neotropical America Section.. Conserv Biol.

[23] Sodhi NS, Posa MRC, Lee TM, Bickford D, Koh LP (2009). The state and conservation of Southeast Asian biodiversity.. Biodivers Conserv.

[24] Balmford A, Whitten T (2003). Who should pay for tropical conservation, and how could the costs be met?. Oryx.

[25] Laurence WF (2004). The perils of payoff: corruption as a threat to global biodiversity.. Trends Ecol Evol.

[26] Cooke WN (2001). The effects of labour costs and workplace constraints on foreign direct investment among highly industrialized countries.. Int J Hum Resour Man.

[27] Konings J, Murphy AP (2006). Do multinational enterprises relocate employment to low-wage regions? Evidence from European multinationals.. Rev World Econ.

[28] Smith RJ, Muir RDJ, Walpole MJ, Balmford A, Leader-Williams N (2003). Governance and the loss of biodiversity.. Nature.

[29] Boyd C, Brooks TM, Butchart SHM, Edgar GJ, da Fonseca GAB (2008). Spatial scale and the conservation of threatened species.. Conserv Lett.

[30] Bottrill M, Joseph LM, Carwardine J, Bode M, Cook C (2008). Is conservation triage just smart decision-making?. Trends Ecol Evol.

[31] Carwadine J, Wilson KA, Ceballos G, Ehrlich PR, Naidoo R (2008). Cost-effective priorities for global mammal conservation.. Proc Natl Acad Sci U S A.

[32] Janicki HP, Wunnava PV (2004). Determinants of foreign direct investment: empirical evidence from EU accession candidates.. J App Econ.

[33] Shaoming C (2006). The role of labour cost in the location choices of Japanese investors in China.. Pap Reg Sci.

[34] Bode M, Wilson KA, Brooks TM, Turner WR, Mittermeier RA (2008). Cost-effective global conservation spending is robust to taxonomic group.. Proc Natl Acad Sci U S A.

[35] Eggera P, Winner H (2005). Evidence on corruption as an incentive for foreign direct investment.. Eur J Polit Econ.

[36] Méon P-G, Sekkat K (2005). Does corruption grease or sand the wheels of growth?. Public Choice.

[37] Rodriguez P, Uhlenbruck K, Eden L (2005). Government corruption and the entry strategies of multinationals.. Acad Manage Rev.

[38] De Sousa L, Larmour P, Hindess B (2009). Governments, NGOs and Anti Corruption: the New Integrity Warrior.

[39] Khawar M (2005). Foreign Direct Investment and Economic Growth: A Cross-Country Analysis.. Global Econ J.

[40] Neumayer E, De Soysa I (2005). Trade openness, Foreign Direct Investment and child labor.. World Dev.

[41] The Alliance for Zero Extinction (2008). Database.. http://www.zeroextinction.org.

[42] UNDP (2008). 2007/2008 Human Development Report: 01 Human development index - GDP index.. http://unstats.un.org/unsd/mdg.

[43] World Bank Group (2000). The World Business Environment Survey.. http://www.gcgf.org/ifcext/economics.nsf/Content/IC-WBESConditions.

[44] The World Bank Group (2010). Worldwide Governance Indicators.. http://info.worldbank.org/governance/wgi/mc_countries.asp.

[45] UNDP (2010). International Human Development Indicators.. http://hdr.undp.org/en/data/trends.

